# Recombineering in *Streptococcus mutans* Using Direct Repeat-Mediated Cloning-Independent Markerless Mutagenesis (DR-CIMM)

**DOI:** 10.3389/fcimb.2017.00202

**Published:** 2017-05-23

**Authors:** Shan Zhang, Zhengzhong Zou, Jens Kreth, Justin Merritt

**Affiliations:** ^1^Department of Restorative Dentistry, Oregon Health and Science UniversityPortland, OR, United States; ^2^State Key Laboratory of Microbial Resources, Institute of Microbiology, Chinese Academy of SciencesBeijing, China; ^3^Department of Molecular Microbiology and Immunology, Oregon Health and Science UniversityPortland, OR, United States

**Keywords:** markerless mutation, negative selection, counterselection, *pheS*, *p*-chlorophenylalanine, *Streptococcus*, natural competence

## Abstract

Studies of the dental caries pathogen *Streptococcus mutans* have benefitted tremendously from its sophisticated genetic system. As part of our own efforts to further improve upon the *S. mutans* genetic toolbox, we previously reported the development of the first cloning-independent markerless mutagenesis (CIMM) system for *S. mutans* and illustrated how this approach could be adapted for use in many other organisms. The CIMM approach only requires overlap extension PCR (OE-PCR) protocols to assemble counterselectable allelic replacement mutagenesis constructs, and thus greatly increased the speed and efficiency with which markerless mutations could be introduced into *S. mutans*. Despite its utility, the system is still subject to a couple limitations. Firstly, CIMM requires negative selection with the conditionally toxic phenylalanine analog *p*-chlorophenylalanine (4-CP), which is efficient, but never perfect. Typically, 4-CP negative selection results in a small percentage of naturally resistant background colonies. Secondly, CIMM requires two transformation steps to create markerless mutants. This can be inherently problematic if the transformability of the strain is negatively impacted after the first transformation step, which is used to insert the counterselection cassette at the mutation site on the chromosome. In the current study, we develop a next-generation counterselection cassette that eliminates 4-CP background resistance and combine this with a new direct repeat-mediated cloning-independent markerless mutagenesis (DR-CIMM) system to specifically address the limitations of the prior approach. DR-CIMM is even faster and more efficient than CIMM for the creation of all types of deletions, insertions, and point mutations and is similarly adaptable for use in a wide range of genetically tractable bacteria.

## Introduction

A large number of medically significant *Streptococcus* species are highly amenable to genetic manipulation, which has been a tremendous boon for our mechanistic understanding of their prominent roles as both human commensals and pathogens. Over the years, the genetic systems for many of these species, such as *Streptococcus mutans*, have been continually evolving to improve upon their ease of use and efficacy, and have now reached the point at which many marked and unmarked mutant strains can be reliably produced from start to finish within a matter of days. Early iterations of the *S. mutans* genetic system relied heavily upon insertion-duplication mutagenesis with suicide vectors (Kuramitsu, 1993, 2003; Russell, 1994). This approach requires the user to first employ classic cloning methodologies to produce mutagenesis constructs in *Escherichia coli*. For such mutations, construct assembly is the major bottleneck, as cloning is a time-consuming process and can be inherently problematic, particularly with DNA derived from A+T-rich organisms (Godiska et al., 2010; Xie et al., 2011). Upon integration, insertion-duplication mutations are also unstable and require constant selective pressure to prevent spontaneous plasmid excision and a regeneration of the parental genotype (Liu et al., 2014). In addition, these mutations introduce a number of plasmid-encoded genes and regulatory elements onto the bacterial chromosome, which frequently trigger a variety of unanticipated genetic regulatory artifacts within the vicinity of the insertion site (Lee et al., 1998). To circumvent this issue, later generations of *S. mutans* insertion-duplication constructs integrated both positive and negative selection markers (i.e., counterselection) to create markerless mutations (Merritt et al., 2007). This approach requires a slight modification of construct design from a traditional insertion-duplication construct to facilitate a two-step plasmid insertion and excision procedure that can generate up to 50% of the recombinants with a desired markerless mutant genotype (Wu and Kaiser, 1996). Since the remaining ≥50% of clones contain the parental genotype, it is also necessary to either PCR-amplify or sequence the clones to identify the mutants. This approach can be used to create all types of deletions, insertions, and point mutations because the mutant strains are free of all chromosomal scars or any other unwanted foreign genetic elements that may otherwise alter the phenotype of the mutant strain. In addition, any number of markerless mutations can be introduced into a single strain, since each round of mutagenesis removes the antibiotic marker. Only one positive and negative selection marker is all that is ever required.

An inherent limitation of markerless mutagenesis in most bacteria is that only a few negative selection markers are available. In species that are unable to metabolize sucrose polymers or galactose, genes encoding levansucrase (*sacB*) and galactokinase (*galK*) can be introduced as negative selection markers (Ueki et al., 1996; Wu and Kaiser, 1996). Unfortunately, many species, such as the streptococci, already encode these genes, which severely limits their utility for negative selection. More recently, we and others have developed negative selection markers using a mutant form of the highly conserved *pheS* gene encoding the alpha subunit of the phenylalanyl-tRNA synthetase (Kristich et al., 2007; Barrett et al., 2008; Xie et al., 2011; Carr et al., 2015; Zhou et al., 2015, 2017; Gurung et al., 2016; Kino et al., 2016; Argov et al., 2017). By engineering a glycine missense mutation into a stringently conserved alanine codon of *pheS* (A314G in *S. mutans*), the mutant gene can be used as a selection marker to sensitize a wild-type strain to the toxic effects of the phenylalanine analog *p*-chlorophenylalanine (4-CP) (Kast and Hennecke, 1991). The only potential limitation of this approach is that some species like *S. mutans* require their own unique mutant *pheS* marker derived from the endogenous wild-type copy of the gene (Xie et al., 2011). Presumably, this is because the mutant PheS protein encoded by the counterselection cassette must compete with the wild-type version to form heteromeric complexes with the beta subunit of the phenylalanyl-tRNA synthetase holoenzyme (Mermershtain et al., 2011). However, the strict conservation of *pheS* among prokaryotes suggests this approach is likely to function in most bacteria, unlike the other commonly employed negative selection markers (Kristich et al., 2007; Xie et al., 2011).

In an effort to increase the speed and simplicity of markerless mutagenesis, we previously developed the first selectable cloning-independent markerless mutagenesis system (CIMM). The CIMM approach completely abrogated the requirement for *E. coli* to serve as an intermediate host for construct assembly (Xie et al., 2011). The key to this approach is the inclusion of a dual selection cassette we referred to as IFDC2, which contains both an antibiotic resistance gene as well as a mutant *pheS* gene encoding the A314G missense mutation. To create markerless mutations, two allelic replacement mutagenesis constructs are assembled using overlap extension PCR (OE-PCR) protocols and then transformed sequentially. The first transformation inserts the cassette into the mutation site using positive selection, while the second transformation excises the cassette using negative selection (Xie et al., 2011). Since all CIMM constructs are created using solely PCR, the entire procedure is extremely rapid and circumvents any potential construct toxicity issues in *E. coli*. The other advantage is that 100% of the transformants contain the desired markerless mutant genotype. The effectiveness of our CIMM approach was recently replicated in *Streptococcus sanguinis* (Gurung et al., 2016). While this system vastly increased the ease and speed with which markerless mutations could be introduced, we still encountered a couple limitations to the system. Firstly, 4-CP negative selection is adequate, but never perfect, as a small, but variable fraction of 4-CP-resistant background clones is typically found on the 4-CP transformation plates. For this reason, it is typically necessary to patch clones from the 4-CP plates onto antibiotic plates to exclude those clones having the parental antibiotic-resistant phenotype. Secondly, the requirement for two transformations can be inherently problematic if the insertion of a counterselection cassette onto the chromosome disrupts a gene required for transformability. In such cases, it is difficult, if not impossible, to remove a cassette like IFDC2 using a second round of transformation. Thus, in the current study, we describe the newest iteration of our cloning-independent markerless mutagenesis system, which addresses the main limitations of the previous version. This new approach, termed DR-CIMM, should also be adaptable for use in many other species for highly efficient and facile markerless mutagenesis.

## Materials and methods

### Primers, bacterial strains, and growth conditions

The primers and bacterial strains used in this study are shown in Tables 1, 2, respectively. *S. mutans* strain UA140 was grown in Todd-Hewitt medium (Difco) supplemented with 0.3% (wt/vol) yeast extract (THYE) or on THYE agar plates. *S. mutans* strains were grown anaerobically (in an atmosphere consisting of 85% N_2_, 10% CO_2_, and 5% H_2_) at 37°C. DNA constructs were introduced into *S. mutans* using natural transformation (Salvadori et al., 2017). Briefly, UA140 and its derivatives were diluted 1:30 from overnight cultures and grown to an optical density of OD_600_ ~0.2 in THYE before the addition of 500 ng ml^−1^ overlap extension PCR (OE-PCR) DNA and 1 μg ml^−1^ CSP. The cultures were subsequently incubated for an additional 2 h and then plated on selective media. For the selection of antibiotic-resistant colonies, THYE plates were supplemented with 15 μg ml^−1^ erythromycin (MP Biomedicals). For counterselection, THYE plates were supplemented with 0.4% *p*-chlorophenylalanine (4-CP, Sigma), unless otherwise stated.

**Table 1:** **Primers used in this study**.

**Primer**	Sequence (5′→3′)^*^	**Purpose**
mubKupF	GAGTATATCATTCAATATATCCTG	Comparison of IFDC2 with ePheS variants to create IFDC3
mubKupR	**TGCTATGAGTGTTATTGTTGCTCGG**CATCCCTCCCACCTAAATATGATTC	
mubKdnF	**TATTAGGTATACTACTGACAGCTTC**TGAAATGAGTGAAAAGGAAATGTCA	
mubKdnR	GACTCGAATTCCATGTGTTTTCTCC	
IFDCF	CCGAGCAACAATAACACTCATAGCA	
IFDCR	GAAGCTGTCAGTAGTATACCTAATA	
IFDC3-T260SF	GCGTCCTTCTTATTTCCCATTT*TCT*GAGCCTTCTGTTGAAGTCGATG	
IFDC3-T260SR	CATCGACTTCAACAGAAGGCTC*AGA*AAATGGGAAATAAGAAGGACGC	
IFDC3-T260AF	GCGTCCTTCTTATTTCCCATTT*GCT*GAGCCTTCTGTTGAAGTCGATG	
IFDC3-T260AR	CATCGACTTCAACAGAAGGCTC*AGC*AAATGGGAAATAAGAAGGACGC	
mub156dnF	**GAATCATATTTAGGTGGGAGGGATG**GGGGCCGCAGAAGCGGAAGGATTAAAAG	Comparison of different direct repeat lengths for deleting 156 bp within the *mubK* gene of the mutanobactin gene locus
mub156dnR	TACTAAATGGTAGCCAATTTGATAG	
DR-R	CATCCCTCCCACCTAAATATGATTC	
DR25IFDC-R	**CATCCCTCCCACCTAAATATGATTC**GAAGCTGTCAGTAGTATACCTAATA	
DR100-F	**TATTAGGTATACTACTGACAGCTTC**CATAAAGTAAGTTCTATTTATTCTG	
DR200-F	**TATTAGGTATACTACTGACAGCTTC**TGGTTGCTTAATGGAATTTCTTGAA	
DR400-F	**TATTAGGTATACTACTGACAGCTTC**ACCAGTTGCTCGCGAACCACCAAGA	
mub625dnF	**GAATCATATTTAGGTGGGAGGGATG**TTGTCTTGTCTTTAGTAGCTGGCAT	Comparison of different deletion lengths within the mutanobactin gene locus
mub2.5dnF	**GAATCATATTTAGGTGGGAGGGATG**AGAAAACACATGGAATTCGAGTCAA	
mub10dnF	**GAATCATATTTAGGTGGGAGGGATG**GTCAAAAGTTTTGACCATGTTGTTCT	
mub4dnF	**GAATCATATTTAGGTGGGAGGGATG**AATAACTATGGGAAGCCAAGAGTAA	
ComYATAAupF	CCAGGCATTCTTATGGATATTGCCG	Creation of markerless *comYA*-TAA nonsense mutation
ComYATAAupR	**TGCTATGAGTGTTATTGTTGCTCGG***TTA*AACCATAAAACCTCCCATCTTATC	
ComYATAADR-F	**TATTAGGTATACTACTGACAGCTTCT**GATACCCCTTTTCGTTCCAAATG	
ComYATAADR-R	**CGGCAATGATTTTTCTACCTAATTT***TTA*AACCATAAAACCTCCCATCTTATC	
ComYATAAdnF	AAATTAGGTAGAAAAATCATTGCCG	
ComYATAAdnR	CTATAATTTCTGTTAAATTAAAACC	
ComYATAGDR-R	**CGGCAATGATTTTTCTACCTAATTT***CTA*AACCATAAAACCTCCCATCTTATC	Creation of markerless *comYA*-TAG nonsense mutation
ComYATAGupR	**TGCTATGAGTGTTATTGTTGCTCGG***CTA*AACCATAAAACCTCCCATCTTATC	
ComYATGADR-R	**CGGCAATGATTTTTCTACCTAATTT***TCA*AACCATAAAACCTCCCATCTTATC	Creation of markerless *comYA*-TGA nonsense mutation
ComYATGAupR	**TGCTATGAGTGTTATTGTTGCTCGG***TCA*AACCATAAAACCTCCCATCTTATC	
comYBTAAupF	GAGAATAGGGAATGAAAGGAGGTTC	Creation of markerless *comYB*-TAA nonsense mutation
ComYBTAAupR	**GCTATGAGTGTTATTGTTGCTCGG***TTA*TCGCATTTTCTTTGTGTATATTTC	
comYBTAADR-F	**TATTAGGTATACTACTGACAGCTTC**GTGGAACAAACAAATTTATGACCTT	
comYBTAADR-R	**CCAAATGCAGACCATTCAACAACCC***TTA*TCGCATTTTCTTTGTGTATATTTC	
ComYBTAAdnF	TAAGGGTTGTTGAATGGTCTGCATTTG	
comYBTAAdnR	CTTTAACCACGGCAGAACCGCCAG	
comYCupF	CACTGAAGGAGAAGCACAAGCAG	Creation of markerless *comYC*-TAA nonsense mutation
ComYCTAADR-R	**CGTACACTGACTCTCTTGATCTT***TTA*CATTATAAATTAACCTCCATATTCTG	
comYCTAAdnF	TAAAAGATCAAGAGAGTCAGTGTACG	
ComYCTAAupR	**CTATGAGTGTTATTGTTGCTCGG***TTA*CATTATAAATTAACCTCCATATTCTG	
comYCDR-F	**TATTAGGTATACTACTGACAGCTTC**AATATGGAGAAGTAAAGTCAAAATTG	
comYCTAAdnR	CAGATACACTACCAGCAATGACAAG	

* *Complementary sequences used for OE-PCR are shown in bold, while mutagenic sequences used to introduce missense and nonsense mutations are underlined in italics*.

**Table 2:** **Strains used in this study**.

**Strains**	Characteristic(s)^*^	**Comments**
UA140	Wild-type *Streptococcus mutans*	Wild type
IFDC2	UA140, IFDC2 cassette insertion in *mubK*, Em^r^, 4-CP^s^	*pheS* A314G
T260A	UA140, IFDC3 cassette insertion in *mubK*, Em^r^, 4-CP^s^	*pheS* A314G/T260A
T260S	UA140, IFDC3 cassette insertion in *mubK*, Em^r^, 4-CP^s^	*pheS* A314G/T260S
Mub156	UA140, markerless deletion in *mub* locus, Em^s^, 4-CP^r^	156 bp deleted after *mubK* start codon
Mub625	UA140, markerless deletion in *mub* locus, Em^s^, 4-CP^r^	625 bp deleted after *mubK* start codon
Mub2.5	UA140, markerless deletion in *mub* locus, Em^s^, 4-CP^r^	2.5 kb deleted after *mubK* start codon
Mub10	UA140, markerless deletion in *mub* locus, Em^s^, 4-CP^r^	10 kb deleted after *mubK* start codon
Mub40	UA140, markerless deletion in *mub* locus, Em^s^, 4-CP^r^	40 kb deleted after *mubK* start codon
YA-TAA	UA140, markerless nonsense mutation in *comYA*, Em^s^, 4-CP^r^	*comYA*, 3rd codon, CAA→TAA
YA-TAG	UA140, markerless nonsense mutation in *comYA*, Em^s^, 4-CP^r^	*comYA*, 3rd codon, CAA→TAG
YA-TGA	UA140, markerless nonsense mutation in *comYA*, Em^s^, 4-CP^r^	*comYA*, 3rd codon, CAA→TGA
YB-TAA	UA140, markerless nonsense mutation in *comYB*, Em^s^, 4-CP^r^	*comYB*, 3rd codon, CAA→TAA
YC-TAA	UA140, markerless nonsense mutation in *comYC*, Em^s^, 4-CP^r^	*comYC*, 2nd codon, AAA→TAA

* *Em, erythromycin; 4-CP, p-chlorophenylalanine*.

### General DNA manipulation

Phusion DNA polymerase (NEB) was used to amplify individual PCR fragments, while AccuPrime Polymerase (Invitrogen) was used for OE-PCR reactions. Taq DNA polymerase (NEB) was used for PCR screening of clones.

### Generation of IFDC3 strains

In order to increase the sensitivity of *S. mutans* to 4-CP when grown on complex media, IFDC3 was generated by introducing an additional mutation (either T260A or T260S) into the IFDC2 cassette (a pheS^*^-*ermAM* two-gene operon driven by the constitutive *ldh* promoter) (Xie et al., 2011). First, the IFDC2 cassette was inserted into the *mubK* gene, which is the first gene in the mutanobactin locus (Wu et al., 2010). A 1.1 kb region upstream of *mubK* was PCR amplified with the primer pair mubKupF and mubKupR, while an 800 bp region downstream of *mubK* was PCR amplified with the primer pair mubKdnF and mubKdnR. The IFDC2 cassette was PCR amplified with the primer pair IFDCF and IFDCR. The three amplicons contain complementary regions, which allowed their subsequent ligation using OE-PCR with the primer pair mubKupF and mubKdnR. The resulting OE-PCR product was transformed into UA140 and selected on a THYE plate containing erythromycin. Next, gDNA was extracted from an IFDC2 transformant and used as a template to create the IFDC3 strains (IFDC3-T260S and IFDC3-T260A) by engineering an additional point mutation (T260S or T260A) into the *pheS* gene of the IFDC2 cassette. A 2.1 kb upstream homologous fragment was PCR amplified with the primer pair mubKupF and IFDC3-T260SR, while a 1.9 kb downstream homologous fragment was PCR amplified with the primer pair IFDC3-T260SF and mubKdnR. Complementary regions of the two amplicons contained the T260S mutation, which facilitated their subsequent OE-PCR ligation using the primer pair mubKupF and mubKdnR. The overlap PCR product containing the T260S mutation was transformed into UA140 and selected on a THYE plate containing erythromycin. The resulting strain IFDC3-T260S was confirmed by PCR and sequencing. The IFDC3-T260A strain was obtained using the same approach, except the primer pair IFDC3-T260AF and IFDC3-T260AR was used.

### Direct repeat-mediated cloning-independent markerless mutagenesis (DR-CIMM)

To avoid interference from the direct repeats during OE-PCR assembly, mutagenesis constructs were created in two separate smaller segments and then transformed simultaneously into *S. mutans* to generate the allelic replacement mutants. To generate the first segment, a 1.1 kb region upstream of *mubK* was PCR amplified with the primer pair mubKupF and mubKupR. The IFDC3 cassette was PCR amplified with primer pair IFDC-F and IFDC-R. The two amplicons contain regions of complementarity, which allowed their subsequent OE-PCR ligation using the primer pair mubKupF and IFDC-R. For the second segment, a 200 bp direct repeat was PCR amplified with primers DR200-F and DR-R, while a 1 kb region downstream of *mubK* was PCR amplified with the primer pair mub156dnF and mub156dnR. The resulting amplicons were mixed with an IFDC3 amplicon and assembled using OE-PCR and the primer pair IFDCF and mub156dnR. The two OE-PCR segments were simultaneously transformed into UA140 and selected on a THYE plate containing erythromycin. Antibiotic resistant transformants were cultured in THYE medium lacking antibiotics and grown to stationary phase. 200 μl of the culture was washed in an equal volume of PBS to remove residual carryover nutrients and then spotted onto 4-CP agar plates in successive dilutions. 4-CP resistant colonies were confirmed by PCR and/or sequencing.

### Comparison of different direct repeats lengths for DR-CIMM

A variety of direct repeat lengths were tested for their utility for DR-CIMM. To create 25 bp direct repeats, the repeat sequence was inserted on the 3'-terminus of the IFDC3 cassette by PCR amplification with the primer pair IFDCF and DR25IFDC-R and on the 5'-terminus of the downstream homologous fragment with the primer pair mub156dnF and mub156dnR. These amplicons were mixed with a previously amplified 1.1 kb *mubK* upstream fragment and assembled using OE-PCR with the primer pair mubKupF and mub156dnR. The resulting PCR product was transformed into UA140. The 100 bp and 400 bp direct repeat constructs were assembled as previously described for the 200 bp direct repeat construct. The respective primer pairs are listed in Table 1.

### Comparison of different deletion lengths for DR-CIMM

A variety of deletions were engineered into the mutanobactin gene locus, each increasing in size by 4-fold from 156 bp up to 40 kb. Constructs were assembled using the previously described OE-PCR methodology and 100 bp direct repeats. The respective primer pairs are listed in Table 1.

### Engineering nonsense point mutations into the *comY* operon

Markerless nonsense point mutations were engineered into several genes of the *comY* operon. The third codon of *comYA* (CAA) was mutated to TAA, TAG, and TGA, while the third codon of *comYB* (CAA) and the second codon of *comYC* (AAA) were both mutated to TAA. Each of the mutations was introduced using the previously described DR-CIMM methodology with primers incorporating the desired point mutations. The respective primer pairs are listed in Table 1. The natural competence of the resulting *comY* mutants was measured by natural transformation of 2 μg ml^−1^ of the *E. coli-Streptococcus* shuttle plasmid pDL278 (Leblanc et al., 1992).

## Results

### Creation of IFDC3, the next-generation counterselection cassette

**Figure 1:**
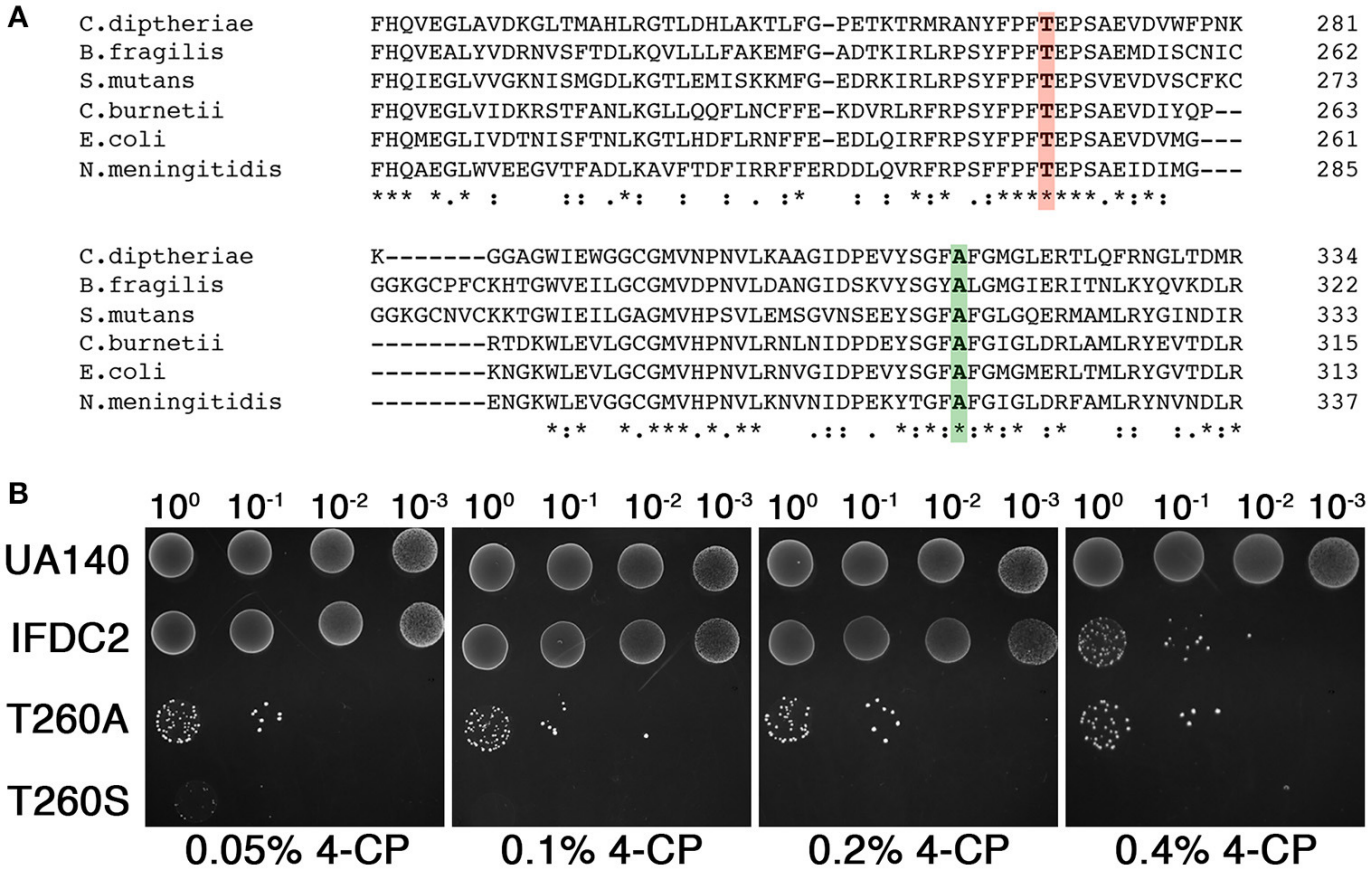
**ePheS negative selection in *S. mutans***. **(A)** The Clustal Omega (Sievers et al., 2011) webserver (http://www.ebi.ac.uk/Tools/msa/clustalo/) was used to align the PheS proteins from a phylogenetically diverse subset of Gram positive and Gram negative bacteria. Residues highlighted in green correspond to A294 in *E. coli* and A314 in *S. mutans*. Residues highlighted in red correspond to T251 in *E. coli* and T260 in *S. mutans*. **(B)** The two *S. mutans* ePheS variants (T260A/A314G and T260S/A314G) were compared to IFDC2 (PheS A314G) for their performance over a range of 4-CP concentrations. The strains from top to bottom are: wild-type (UA140), A314G *pheS* insertion (IFDC2), T260A/A314G *epheS* insertion (T260A), and T260S/A314G *epheS* insertion (T260S).

While 4-CP negative selection has proven to be a major advance for markerless mutagenesis in numerous species, multiple studies have reported a small percentage of 4-CP resistant background clones arising after negative selection, especially when using complex media (Kristich et al., 2007; Xie et al., 2011; Miyazaki, 2015; Argov et al., 2017). We have consistently encountered a similar phenomenon with *S. mutans*, making it necessary to screen 4-CP resistant clones to identify the desired markerless mutants. Recently, a new *E. coli* PheS variant referred to as ePheS was reported to exhibit substantially increased sensitivity to 4-CP when grown on complex medium (Miyazaki, 2015). ePheS contains the original A294G substitution required for 4-CP selection in *E. coli* in addition to either a T251A or a T251S mutation. Since both of the T251A/A294G and T251S/A294G ePheS mutants exhibited stringent negative selection in the presence of 4-CP, we were curious to test whether these mutations would similarly improve 4-CP selection in *S. mutans*. To identify the equivalent residue in the *S. mutans* PheS, we performed a multiple sequence alignment of PheS proteins from a phylogenetically diverse group of bacteria. Consistent with previous observations, alanine 294 from the *E. coli* PheS is strictly conserved in the alignment (Figure 1A), despite considerable sequence divergence overall (Kristich et al., 2007; Xie et al., 2011). Likewise, threonine 251 is centered within a cluster of conserved residues (Figure 1A), indicating that this region of PheS is likely to be critical for its functionality in the vast majority of bacteria. Next, we engineered both T260A and T260S mutations into the A314G PheS protein encoded by the IFDC2 cassette and then compared their performance to that of IFDC2. Similar to the results reported in *E. coli*, both of the T260 mutations exhibited synergistic effects when combined with the A314G mutation (Figure 1B). However, in contrast to *E. coli*, we observed much greater selectivity with the T260S mutation compared to the T260A variant (Figure 1B). In fact, the T260S/A314G ePheS completely inhibited all background growth in as little as 0.1% (wt/vol) 4-CP, which is 4-fold lower than what is normally required for selection with the IFDC2 cassette (Figure 1B). At 0.05% (wt/vol) 4-CP, we did observe extremely small colonies on the plates, indicating that the cells were not fully inhibited. Thus, 0.1% (wt/vol) 4-CP is the minimal effective concentration required for ePheS negative selection in *S. mutans*.

### Direct repeat-mediated cloning-independent markerless mutagenesis (DR-CIMM)

**Figure 2:**
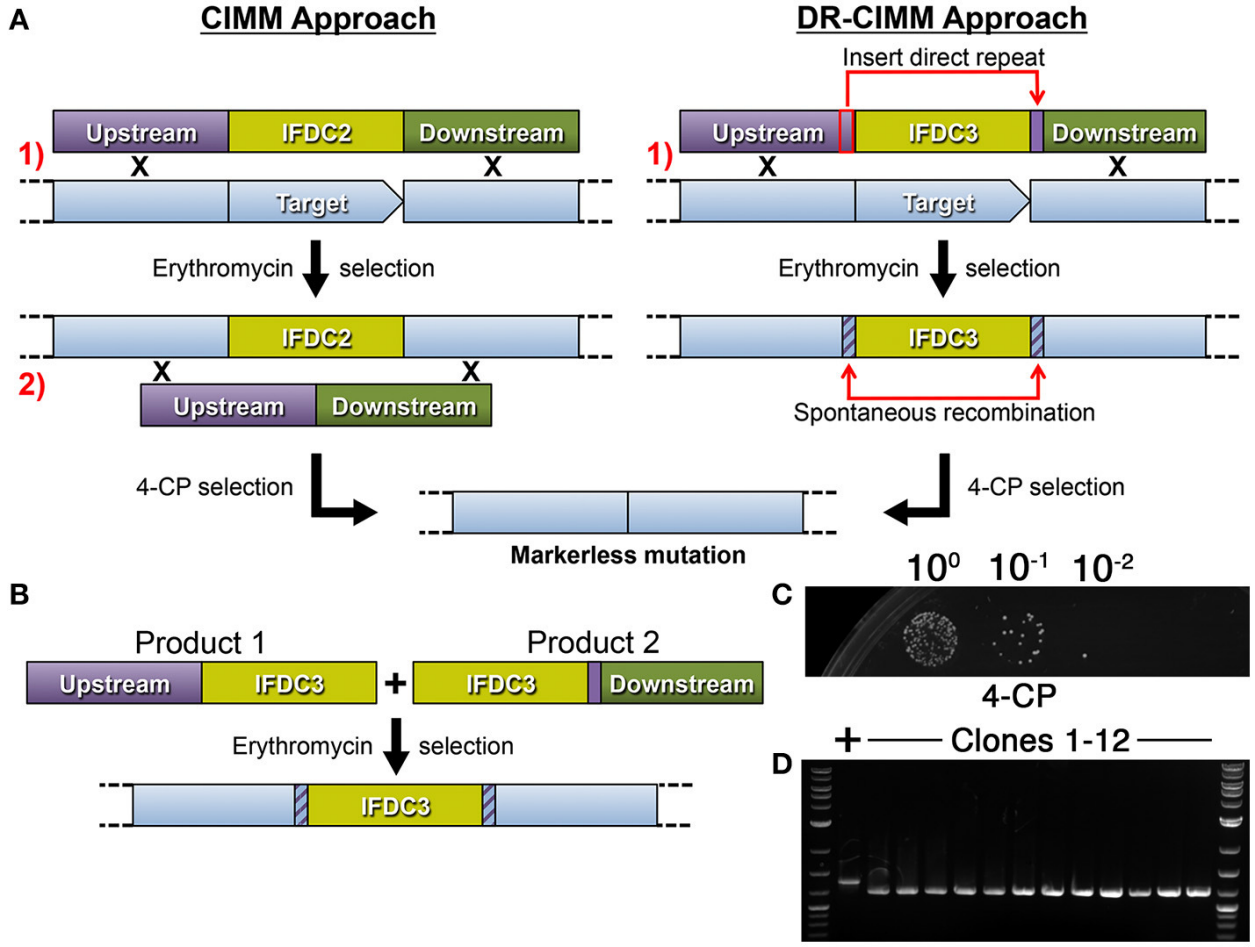
**Direct repeat-mediated cloning-independent markerless mutagenesis (DR-CIMM)**. **(A)** Comparison of the CIMM and DR-CIMM approaches. Individual transformation steps are numbered in red. The previous IFDC2-based CIMM approach (shown on the left) requires two sequential transformations with two separate OE-PCR products. The first transformation inserts the IFDC2 cassette onto the chromosome via double crossover homologous recombination and transformants are selected based upon acquired erythromycin resistance. The second transformation excises the IFDC2 cassette to render the cells 4-CP-resistant. The current IFDC3-based DR-CIMM approach (shown on the right) requires a single transformation with an OE-PCR product. The mutagenesis construct contains a small segment of the 3' end of the upstream homologous fragment (outlined in red) inserted immediately after the IFDC3 cassette, which serves as the direct repeat. After transformation and selection on erythromycin, clones are cultured in non-selective medium to excise the cassette due to spontaneous recombination between the two direct repeats. These clones are subsequently isolated due to their acquired 4-CP resistance. **(B)** Illustration of the two-fragment DR-CIMM construct assembly approach. To avoid interference from the direct repeats during OE-PCR assembly, two smaller segments of the final DR-CIMM construct are assembled by OE-PCR, separating the direct repeats between the two fragments. The first OE-PCR product is created by attaching the IFDC3 cassette onto the 3' of the upstream homologous fragment, while the second OE-PCR product is created by attaching the direct repeat and downstream homologous fragment onto the 3' of IFDC3 cassette. Thus, both OE-PCR products contain a copy of IFDC3. The two OE-PCR products are transformed simultaneously into *S. mutans* and selected for antibiotic resistance. Homologous recombination between the OE-PCR products assembles the final construct *in vivo*, which is then recombined with the chromosome. **(C)** A 156 bp deletion construct was created using IFDC3 and the previously described DR-CIMM methodology. Shown here are the results obtained from the final 4-CP negative selection step. **(D)** 12 CFU were randomly selected from the 4-CP plates and PCR-amplified to compare their genotypes. All clones exhibited the expected 156 bp deletion, resulting in smaller PCR amplicons relative to the parent wild-type (+).

By incorporating *epheS* into our counterselection cassette (now referred to as IFDC3), we effectively eliminated all detectable background growth during 4-CP selection. Our next goal was to circumvent the requirement for two transformation steps for cloning-independent markerless mutagenesis. In the model organism *Saccharomyces cerevisiae*, a markerless mutagenesis approach termed mutagenic inverted repeat assisted genome engineering (MIRAGE) was developed to increase the speed and efficiency of markerless mutagenesis (Nair and Zhao, 2009). For this approach, an inverted repeat of a *ura3* selection marker is inserted between two 25 bp direct repeats derived from the target mutation site. The presence of the inverted repeat stimulates recombination between the two 25 bp direct repeats flanking the cassette, ultimately resulting in its excision and a markerless mutation. Much of the MIRAGE construct can be assembled by OE-PCR and only a single transformation step is required. However, it is necessary to employ restriction digestion/ligation to assemble the *ura3* inverted repeat, due to its interference with OE-PCR. Thus, the procedure is not entirely cloning-independent. To simplify construct assembly, we hypothesized that it would be possible to forgo the inverted repeat segment and generate the desired markerless mutants simply by creating direct repeats flanking either side of IFDC3. In this case, the overall approach would be similar to CIMM, but would only require a single transformation (Figure 2A). To test this, we designed a 4-piece OE-PCR deletion construct containing a 200 bp direct repeat duplicated from the upstream homologous fragment and placed after the 3' terminus of the IFDC3 cassette (Figure 2A). Unfortunately, we found the presence of the direct repeat to be a major hindrance for OE-PCR assembly. Presumably, the direct repeats serve as complementary ends that compete with the complementary ends of the OE-PCR fragments and ultimately inhibit their properly assembly. To mitigate this issue and further simplify construct assembly, we separated the direct repeats onto two smaller OE-PCR segments of the final construct, including a copy of the IFDC3 cassette on each (Figure 2B). Next, we simultaneously transformed both OE-PCR amplicons directly into *S. mutans* and amazingly observed that the final step of construct assembly could be performed *in vivo* simply using the homologous recombination system of the cell. Thus, it was possible to reduce the difficult 4-piece OE-PCR reaction into two smaller, easily assembled OE-PCR reactions. Using this assembly approach, we reliably obtained a large number of erythromycin resistant clones, several of which were inoculated into non-selective medium, grown to stationary phase, and then dilutions were spotted onto 4-CP plates. Afterward, numerous 4-CP resistant clones were detectable and all contained the expected 156 bp deletion (Figures 2C,D), indicating a lack of background growth and a precise excision of the IFDC3 cassette. Since this approach seemed highly efficient for both construct assembly and 4-CP selection, all subsequent reactions were performed similarly.

### Effect of direct repeat length and deletion length on mutagenesis efficiency

**Figure 3:**
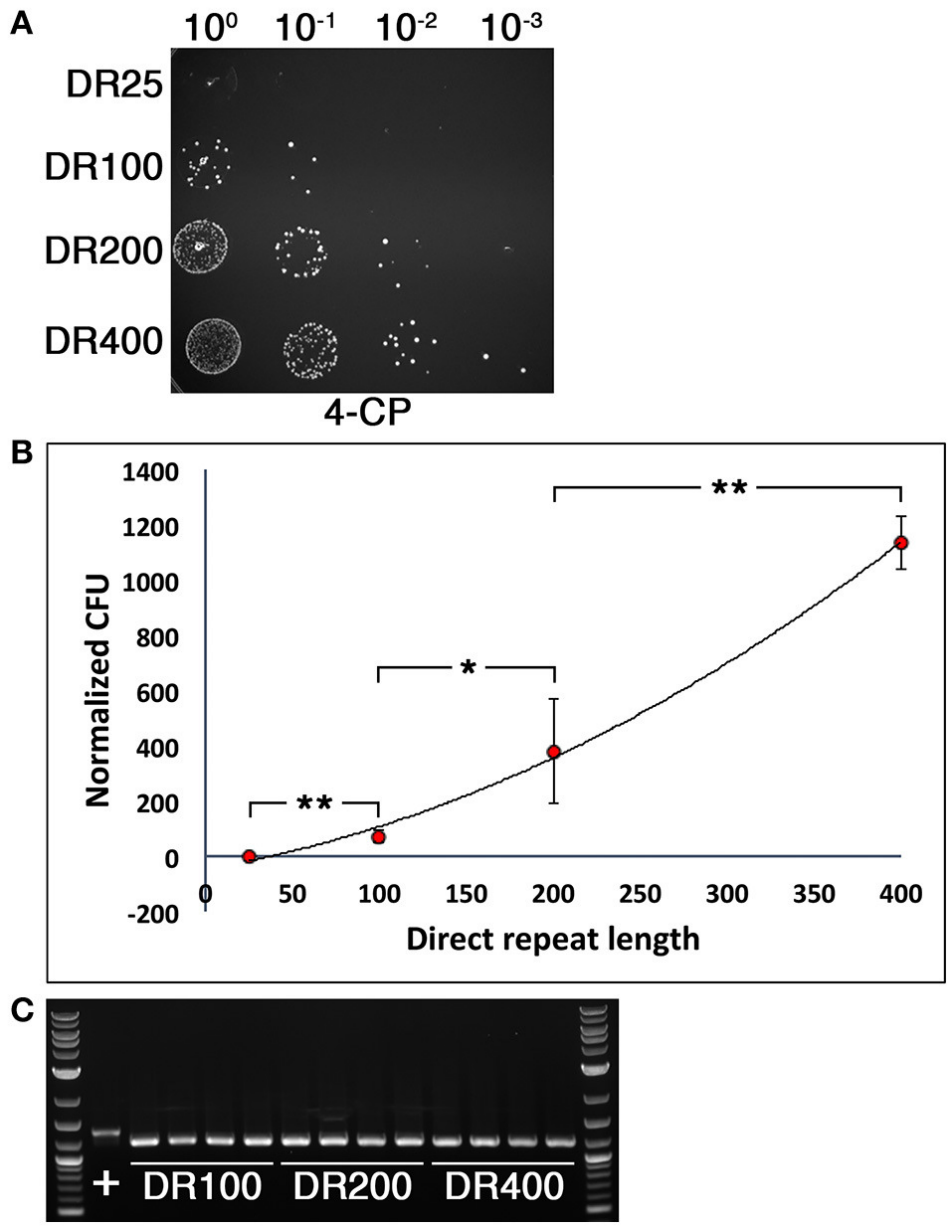
**Influence of direct repeat length upon markerless mutagenesis efficiency**. **(A)** Four separate 156 bp deletion constructs were created using IFDC3 and the previously described DR-CIMM methodology. The constructs only varied in the sizes of the inserted direct repeats after the IFDC3 cassette. Shown here are the results obtained from the final 4-CP negative selection step. The strains from top to bottom are: DR25 (25 bp direct repeats), DR100 (100 bp direct repeats), DR200 (200 bp direct repeats), and DR400 (400 bp direct repeats). **(B)** The number of markerless mutant clones obtained using a range of direct repeat lengths was compared. The data are presented as the means ± standard deviations from three independent experiments. ^*^P < 0.05 and ^**^P < 0.01 as determined by unpaired two-tailed *t*-test. **(C)** 4 CFU from the DR100, DR200, and DR400 constructs were randomly selected from the 4-CP plates and PCR-amplified to compare their genotypes. All clones exhibited the expected 156 bp deletion, resulting in smaller PCR amplicons relative to the parent wild-type (+).

We were next interested to determine the optimal direct repeat length for DR-CIMM as well as to assess the impact of chromosomal deletion length upon DR-CIMM efficiency. Targeting the same 156 bp deletion generated in Figure 2D, we compared the number of 4-CP resistant clones obtained with constructs having 25 bp, 100 bp, 200 bp, and 400 bp direct repeats. As expected, increasing the lengths of the direct repeats significantly increased the efficiency of markerless mutagenesis (Figure 3A). While the efficiency of 25 bp direct repeat mutagenesis was below our limit of detection (i.e., no clones), we observed a nearly linear increase in the number of clones obtained from 100 to 400 bp direct repeats (Figure 3B). It was impractical to test constructs with >400 bp direct repeats because beyond this size, unintended allelic replacement mutants arise that use the direct repeat for the initial recombination event, rather than the downstream homologous fragment (data not shown). These improperly targeted clones exclusively yield a wild-type genotype after counterselection, since the IFDC3 cassette is not inserted into its intended location. For the 100–400 bp direct repeat constructs, 100% of the 4-CP-resistant clones yielded the desired markerless deletions (Figure 3C).

**Figure 4:**
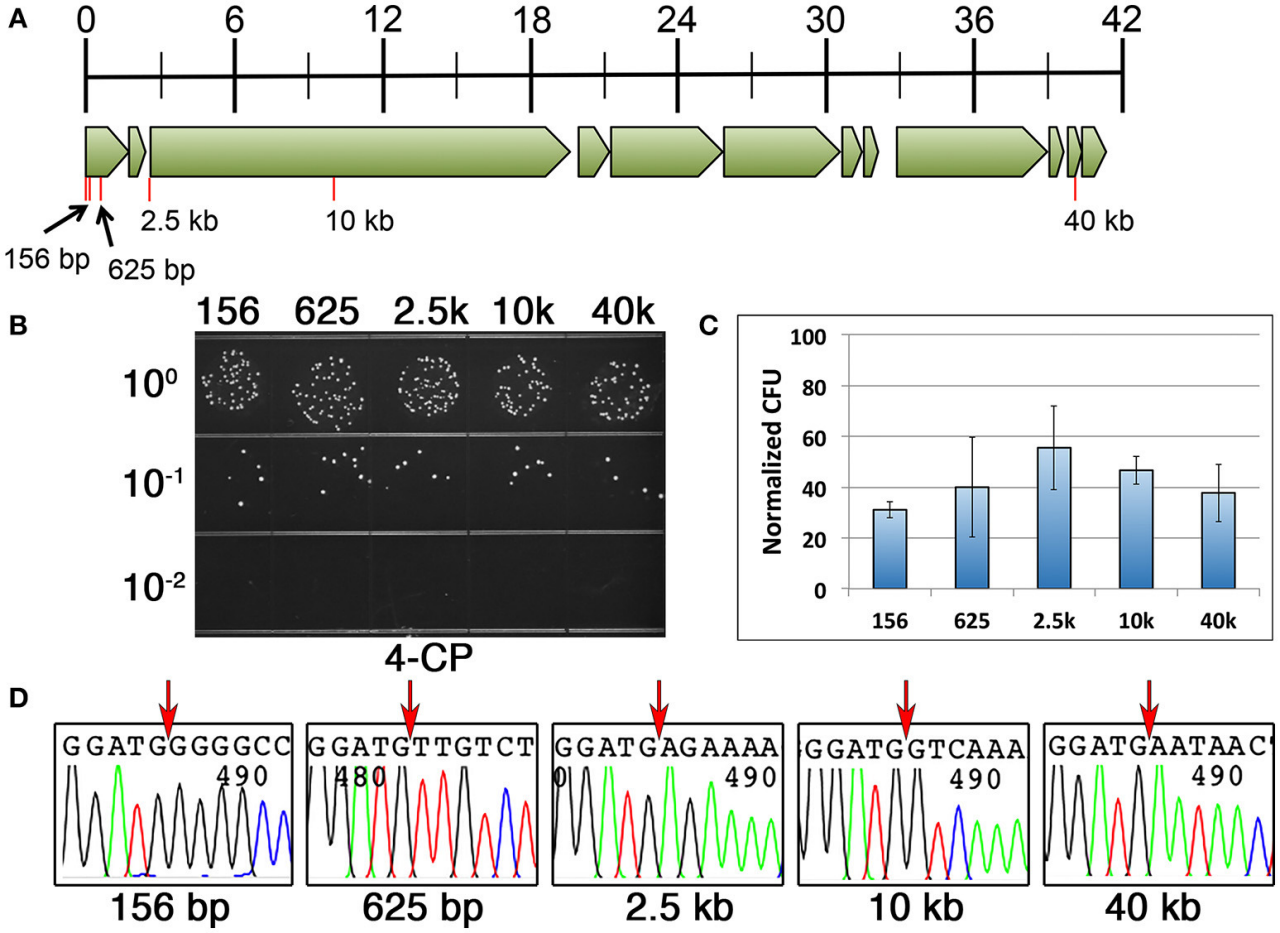
**Influence of deletion size upon markerless mutagenesis efficiency**. **(A)** Genomic map of the UA140 mutanobactin gene locus (Wu et al., 2010). Open reading frames are drawn to scale and tick marks in the scale bar indicate size in kb. Red lines indicate the locations and sizes of the different deletions. All deletions start immediately after the translation start codon of the first open reading frame in the mutanobactin locus (*mubK*). **(B)** 100 bp direct repeats were used for markerless deletions in the mutanobactin locus. Deletions ranged in size from 156 to 40 kb, as indicated above the image. Shown here are the results obtained from the final 4-CP selection step. **(C)** The number of markerless mutant clones obtained from each of the deletion constructs was compared. The data are presented as the means ± standard deviations from three independent experiments. None of the means exhibited statistically significant differences as determined by unpaired two-tailed *t*-tests. **(D)** Multiple markerless deletion mutant clones were sequenced to assess deletion fidelity. Shown here are representative chromatograms from each unique mutant. Red arrows indicate the locations of the deletions immediately following the start codon of the first open reading frame in the mutanobactin locus (*mubK*).

We repeated the same DR-CIMM procedure and compared the mutagenesis efficiencies for markerless chromosomal deletions ranging in size from 156 to 40 kb (Figure 4A). Interestingly, in contrast to our previous results, the 200 and 400 bp direct repeat constructs yielded a portion of the transformants with incorrectly targeted IFDC3 allelic replacements, but only when creating deletions ≥2.5 kb. Fortunately, this was not an issue for any of the deletions when using 100 bp direct repeats. Therefore, we focused our analysis solely upon the 100 bp direct repeat constructs. We were quite surprised to discover that deletion length had no appreciable impact upon recombination efficiency for any of the tested deletions, even up to 40 kb (Figures 4B,C). Thus, for deletions ranging in size from 156 to 40 kb, markerless mutants can be generated with nearly identical efficiencies using 100 bp direct repeats. Once again, we failed to detect any evidence of 4-CP-resistant background growth, as all tested clones had excised the IFDC3 cassette. We also sequenced multiple clones from each of the reactions and all contained precise deletions, indicating that deletion length had no detrimental impacts upon recombination fidelity (Figure 4D).

### Markerless mutagenesis in natural competence-defective mutants

**Figure 5:**
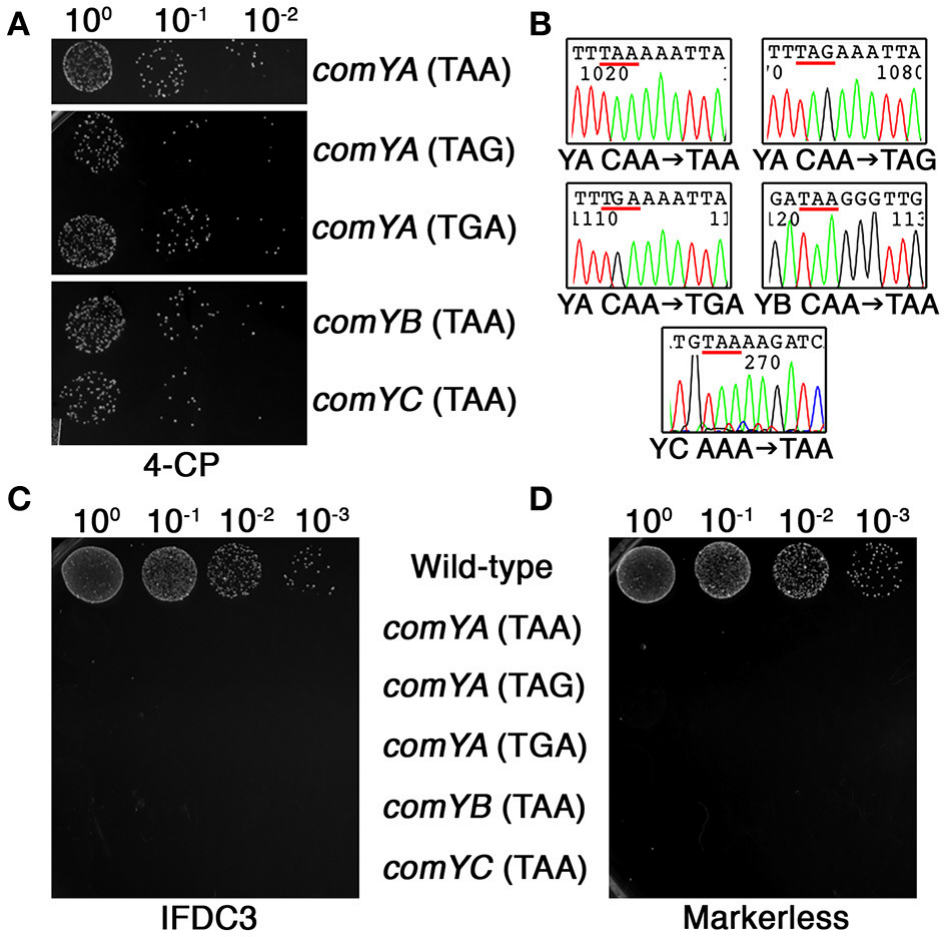
**Markerless nonsense point mutations in the *comY* operon**. **(A)** Multiple markerless nonsense point mutations were introduced into *comYA, comYB*, and *comYC*. Shown here are the results obtained from the final 4-CP selection step. The mutated genes and the sequences of the engineered stop codons are indicated on the right side of the image. **(B)** Multiple markerless nonsense point mutant clones were sequenced to assess mutation fidelity. Shown here are representative chromatograms with the engineered stop codons underlined in red. The identity of the mutated genes (*comYA, YB*, or *YC*) and their engineered point mutations are listed under their corresponding chromatograms. **(C)** Each of the parental allelic replacement mutants containing IFDC3 was tested for their natural competence ability. Shown here are the resulting transformants obtained after transformation with 2 μg ml^−1^ of the *E. coli-Streptococcus* shuttle vector pDL278. Strain identities are indicated on the right side of the image. **(D)** The same transformation experiment was repeated using confirmed markerless nonsense *comY* point mutant strains. Strain identities are indicated on the left side of the image.

As previously mentioned, one of the principal limitations of our previous CIMM system was the requirement for two transformation reactions. The first transformation is required to insert the counterselection cassette, while the second transformation is used to excise it. Difficulties can arise when the first transformation step creates a mutant strain having reduced or defective natural competence development, which then interferes with subsequent transformations. To determine whether our current system could circumvent this limitation, we employed DR-CIMM to engineer a series of markerless nonsense point mutations into the first three genes of the *comY* operon. The *comY* genes encode essential components of the natural competence machinery and defects in these genes render the cell completely resistant to transformation via natural competence (Merritt et al., 2005). Despite this, DR-CIMM functioned flawlessly to create all three types of nonsense point mutations into the *comYA* gene (Figures 5A,B). We also introduced ochre nonsense point mutations into both the *comYB* and *comYC* genes with similar efficiencies and perfect fidelity (Figures 5A,B). Next, we performed transformation assays to test the natural competence abilities of both the parental IFDC3 insertion strains and their resulting markerless point mutants. Insertion of the IFDC3 cassette resulted in natural competence-defective phenotypes for each of the mutants, due to its disruption of *comYA, comYB*, or *comYC* (Figure 5C). Likewise, all five markerless nonsense mutants failed to yield any detectable transformants as well, further indicating the functionality of the engineered stop codons (Figure 5D). Thus, in contrast to the previous CIMM system, excision of the IFDC3 cassette via DR-CIMM occurred both efficiently and precisely in strains having natural competence-negative phenotypes.

## Discussion

In the current study, we describe the latest iteration of our cloning-independent markerless mutagenesis system, which is now nearly as simple and rapid to engineer as more traditional marked mutagenesis approaches. Importantly, the described DR-CIMM methodology should be adaptable for use in many other organisms to facilitate efficient and facile genome editing and recombineering. The only fundamental limitation is the ability to introduce double-crossover allelic replacement mutations, since this approach utilizes OE-PCR products to circumvent the cloning requirement needed for plasmid-based mutagenesis. For organisms with high rates of transformation and recombination like many naturally competent species, it may be similarly feasible to employ the two-fragment construct assembly procedure described in Figure 2B. This greatly simplifies construct assembly, since the individual OE-PCR products are smaller, less complex, and do not contain repeat sequences that can interfere with OE-PCR. We have also found the two-fragment assembly approach to be quite useful for traditional marked allelic replacement mutation constructs, especially for more complex constructs having 4 or more individual fragments.

Since our approach is free of 4-CP resistant background growth and only requires a single transformation, it is feasible to quickly generate markerless mutations without having to screen for clones having the appropriate genotype. For example, markerless mutations can be created in *S. mutans* in the absence of selection simply by stimulating extremely high levels of natural competence and then transforming mutagenesis constructs having ≥2 kb recombination arms (Morrison et al., 2015). In this case, up to 10–50% of the resulting transformants may contain the desired mutation, which necessitates a final screening step to identify the mutants. In some cases, this may be a time-consuming process, particularly when identifying point mutant clones. By incorporating a stringent negative selection marker, a final screening step is unnecessary, as 100% of the transformants should contain the desired genotype. Clones can then be directly sequenced for an independent confirmation. It is worth noting that in our experience, achieving 100% efficiency was contingent upon incorporating the appropriately sized direct repeats in the mutagenesis constructs. For *S. mutans*, ~100 bp direct repeats offers the best combination of efficiency and efficacy, functioning well for all types and sizes of mutations (Figures 4, 5). Larger direct repeats can increase the yield of markerless mutants, but depending upon the size of the engineered mutations, one also runs the risk of having the counterselection cassette insert into the wrong location in a fraction of the transformants. Such clones will regenerate a parental genotype after 4-CP selection. However, even with 100 bp direct repeats, we consistently obtained >4000 CFU ml^−1^ of markerless mutants (Figures 3A, 4B). Thus, there was always a large excess of clones. If for some reason additional clones were required, one could presumably increase the number of generations the IFDC3-containing clones are cultured without selection before plating on 4-CP agar plates. For deletions or insertions smaller than 2.5 kb, 200 bp direct repeats should also increase the yield of mutants without discernable problems. The inclusion of direct repeats in the current system also solved one of the largest hindrances of the previous system: the requirement for two transformation events. Using CIMM, we have encountered numerous instances in which insertion of the IFDC2 cassette resulted in a mutant strain with reduced transformability, making it difficult or impossible to perform a second round of transformation to excise the cassette (unpublished results). However, with DR-CIMM, we were able to engineer multiple nonsense point mutations into several genes of the *comY* operon. Such mutations would have been extremely difficult to engineer using the previous system, as counterselection cassette insertions into these genes result in natural competence-negative phenotypes (Figure 5C). Using the DR-CIMM approach, the *comY* mutants were obtained at comparable rates as mutations engineered within the mutanobactin gene cluster (Figures 3B, 5A), which is not required for natural competence development (Wu et al., 2010). Thus, defects in competence development did not appear to exert any influence upon the ability of the cell to excise IFDC3 through homologous recombination. Consequently, we would expect this system to be similarly useful for many other genetically tractable species including those in which natural transformation is not an option.

## Author contributions

Experiments and data analysis were performed by SZ and ZZ. The manuscript was written by SZ, JK, and JM.

### Conflict of interest statement

The authors declare that the research was conducted in the absence of any commercial or financial relationships that could be construed as a potential conflict of interest.

## References

[B1] Argov T. Rabinovich L. Sigal N. Herskovits A. A. (2017). An effective counterselection system for Listeria monocytogenes and its use to characterize the monocin genomic region of strain 10403S. Appl. Environ. Microbiol. 83:e02927–16. 10.1128/AEM.02927-16 28039138 PMC5335520

[B2] Barrett A. R. Kang Y. Inamasu K. S. Son M. S. Vukovich J. M. Hoang T. T. (2008). Genetic tools for allelic replacement in Burkholderia species. Appl. Environ. Microbiol. 74, 4498–4508. 10.1128/AEM.00531-08 18502918 PMC2493169

[B3] Carr J. F. Danziger M. E. Huang A. L. Dahlberg A. E. Gregory S. T. (2015). Engineering the genome of *Thermus thermophilus* using a counterselectable marker. J. Bacteriol. 197, 1135–1144. 10.1128/JB.02384-14 25605305 PMC4336342

[B4] Godiska R. Mead D. Dhodda V. Wu C. Hochstein R. Karsi A.. (2010). Linear plasmid vector for cloning of repetitive or unstable sequences in *Escherichia coli*. Nucleic Acids Res. 38:e88. 10.1093/nar/gkp1181 20040575 PMC2847241

[B5] Gurung I. Berry J. L. Hall A. M. J. Pelicic V. (2016). Cloning-independent markerless gene editing in *Streptococcus sanguinis*: novel insights in type IV pilus biology. Nucleic Acids Res. 45, e40. 10.1093/nar/gkw1177 27903891 PMC5389465

[B6] Kast P. Hennecke H. (1991). Amino acid substrate specificity of Escherichia coli phenylalanyl-tRNA synthetase altered by distinct mutations. J. Mol. Biol. 222, 99–124. 10.1016/0022-2836(91)90740-W 1942071

[B7] Kino Y. Nakayama-Imaohji H. Fujita M. Tada A. Yoneda S. Murakami K.. (2016). Counterselection employing mutated pheS for markerless genetic deletion in Bacteroides species. Anaerobe 42, 81–88. 10.1016/j.anaerobe.2016.09.004 27639596

[B8] Kristich C. J. Chandler J. R. Dunny G. M. (2007). Development of a host-genotype-independent counterselectable marker and a high-frequency conjugative delivery system and their use in genetic analysis of *Enterococcus faecalis*. Plasmid 57, 131–144. 10.1016/j.plasmid.2006.08.003 16996131 PMC1852458

[B9] Kuramitsu H. K. (1993). Virulence factors of mutans streptococci: role of molecular genetics. Crit. Rev. Oral Biol. Med. 4, 159–176. 10.1177/10454411930040020201 8435464

[B10] Kuramitsu H. K. (2003). Molecular genetic analysis of the virulence of oral bacterial pathogens: an historical perspective. Crit. Rev. Oral Biol. Med. 14, 331–344. 10.1177/154411130301400504 14530302

[B11] Leblanc D. J. Lee L. N. Abu-Al-Jaibat A. (1992). Molecular, genetic, and functional analysis of the basic replicon of pVA380-1, a plasmid of oral streptococcal origin. Plasmid 28, 130–145. 10.1016/0147-619X(92)90044-B 1409970

[B12] Lee M. S. Seok C. Morrison D. A. (1998). Insertion-duplication mutagenesis in Streptococcus pneumoniae: targeting fragment length is a critical parameter in use as a random insertion tool. Appl. Environ. Microbiol. 64, 4796–4802. 9835564 10.1128/aem.64.12.4796-4802.1998 PMC90924

[B13] Liu E. Y. Chang F. Y. Chang J. C. Fung C. P. (2014). Differences in virulence of pneumolysin and autolysin mutants constructed by insertion duplication mutagenesis and in-frame deletion in *Streptococcus pneumoniae*. BMC Biotechnol. 14:16. 10.1186/1472-6750-14-16 24558977 PMC3936844

[B14] Mermershtain I. Finarov I. Klipcan L. Kessler N. Rozenberg H. Safro M. G. (2011). Idiosyncrasy and identity in the prokaryotic Phe-system: crystal structure of E. *coli* phenylalanyl-tRNA synthetase complexed with phenylalanine and AMP. Protein Sci. 20, 160–167. 10.1002/pro.549 21082706 PMC3047072

[B15] Merritt J. Qi F. Shi W. (2005). A unique nine-gene comY operon in *Streptococcus mutans*. Microbiology 151, 157–166. 10.1099/mic.0.27554-0 15632435

[B16] Merritt J. Tsang P. Zheng L. Shi W. Qi F. (2007). Construction of a counterselection-based in-frame deletion system for genetic studies of *Streptococcus mutans*. Oral Microbiol. Immunol. 22, 95–102. 10.1111/j.1399-302X.2007.00329.x 17311632

[B17] Miyazaki K. (2015). Molecular engineering of a PheS counterselection marker for improved operating efficiency in *Escherichia coli*. BioTechniques 58, 86–88. 10.2144/000114257 25652032

[B18] Morrison D. A. Khan R. Junges R. Amdal H. A. Petersen F. C. (2015). Genome editing by natural genetic transformation in *Streptococcus mutans*. J. Microbiol. Methods 119, 134–141. 10.1016/j.mimet.2015.09.023 26481669

[B19] Nair N. U. Zhao H. (2009). Mutagenic inverted repeat assisted genome engineering (MIRAGE). Nucleic Acids Res. 37, e9. 10.1093/nar/gkn943 19050015 PMC2615605

[B20] Russell R. R. (1994). The application of molecular genetics to the microbiology of dental caries. Caries Res. 28, 69–82. 10.1159/000261625 8156565

[B21] Salvadori G. Junges R. Khan R. Amdal H. A. Morrison D. A. Petersen F. C. (2017). Natural transformation of oral Streptococci by use of synthetic pheromones. Methods Mol. Biol. 1537, 219–232. 10.1007/978-1-4939-6685-1_13 27924597

[B22] Sievers F. Wilm A. Dineen D. Gibson T. J. Karplus K. Li W.. (2011). Fast, scalable generation of high-quality protein multiple sequence alignments using clustal omega. Mol. Syst. Biol. 7, 539. 10.1038/msb.2011.75 21988835 PMC3261699

[B23] Ueki T. Inouye S. Inouye M. (1996). Positive-negative KG cassettes for construction of multi-gene deletions using a single drug marker. Gene 183, 153–157. 10.1016/S0378-1119(96)00546-X 8996101

[B24] Wu C. Cichewicz R. Li Y. Liu J. Roe B. Ferretti J.. (2010). Genomic island TnSmu2 of *Streptococcus mutans* harbors a nonribosomal peptide synthetase-polyketide synthase gene cluster responsible for the biosynthesis of pigments involved in oxygen and H_2_O_2_ tolerance. Appl. Environ. Microbiol. 76, 5815–5826. 10.1128/AEM.03079-09 20639370 PMC2935078

[B25] Wu S. S. Kaiser D. (1996). Markerless deletions of pil genes in *Myxococcus xanthus* generated by counterselection with the *Bacillus subtilis* sacB gene. J. Bacteriol. 178, 5817–5821. 10.1128/jb.178.19.5817-5821.1996 8824635 PMC178429

[B26] Xie Z. Okinaga T. Qi F. Zhang Z. Merritt J. (2011). Cloning-independent and counterselectable markerless mutagenesis system in *Streptococcus mutans*. Appl. Environ. Microbiol. 77, 8025–8033. 10.1128/AEM.06362-11 21948849 PMC3208986

[B27] Zhou C. Shi L. Ye B. Feng H. Zhang J. Zhang R.. (2017). pheS^*^, an effective host-genotype-independent counter-selectable marker for marker-free chromosome deletion in *Bacillus amyloliquefaciens*. Appl. Microbiol. Biotechnol. 101, 217–227. 10.1007/s00253-016-7906-9 27730334

[B28] Zhou P. Li X. Qi F. (2015). Establishment of a counter-selectable markerless mutagenesis system in Veillonella atypica. J. Microbiol. Methods 112, 70–72. 10.1016/j.mimet.2015.03.010 25771833 PMC4388892

